# Signet-ring cell melanoma with sentinel lymph node metastasis: A case report with immunohistochemical analysis and review of the clinicopathological features

**DOI:** 10.3892/ol.2013.1669

**Published:** 2013-11-07

**Authors:** MITSUAKI ISHIDA, MUNEO IWAI, KEIKO YOSHIDA, AKIKO KAGOTANI, HIDETOSHI OKABE

**Affiliations:** Department of Clinical Laboratory Medicine and Division of Diagnostic Pathology, Shiga University of Medical Science, Otsu, Shiga 520-2192, Japan

**Keywords:** signet-ring cell melanoma, intermediate filament, mTOR pathway

## Abstract

Signet-ring cell melanoma is an extremely rare variant of malignant melanoma. A 68-year-old male presented with a black nodule on the left thigh. Histopathological examination revealed proliferation of sheet-like or variable-sized nests of atypical melanocytes. Neoplastic cells showing signet-ring cell appearance, characterized by the presence of eccentrically located enlarged nuclei and abundant pale cytoplasm, were also present. Immunohistochemically, the tumor cells were positive for S-100 protein, vimentin and Melan-A. Moreover, mammalian target of rapamycin (mTOR) pathway proteins were diffusely expressed. The current case report presents the 21st reported case of signet-ring cell melanoma. Analyses of the clinicopathological features revealed that this disease commonly affects middle-aged males and the presence of metastatic signet-ring cell melanoma with an unknown primary tumor. Immunohistochemical analyses of melanocytic markers have been useful for establishing the diagnosis of this type of disease, however, HMB-45 is occasionally found to be negative. In addition, the present case report is the first to analyze the expression of mTOR pathway proteins, which are central proteins involved in carcinogenesis and its inhibitor has been proposed as a therapeutic target for various types of tumor. Therefore, the mTOR inhibitor may also be a potential candidate for the treatment of this type of tumor.

## Introduction

Malignant melanoma occasionally shows a variety of cytomorphological and architectural features, including balloon, rhabdoid, small, myxoid, adenoid (pseudoglandular) and clear cell types (1). Signet-ring cell melanoma is one of the rarest histopathological variants of malignant melanoma, characterized histopathologically by the presence of tumor cells in which the nucleus is compressed to the cellular periphery, appearing as signet-rings (2). This variant was initially described by Sheibani and Battifora in 1988 (3) and since then, 20 cases have been reported in English literature (2–15). The current case report presents an additional case of signet-ring cell melanoma with sentinel lymph node metastasis, analyses of the immunohistochemical expression profiles of the intermediate filaments and mammalian target of rapamycin (mTOR) pathway proteins and review of the clinicopathological features of this extremely rare variant of malignant melanoma.

## Case report

### Patient presentation

A 68-year-old male without a past history of malignant melanoma presented with a gradually enlarged black nodule in the left thigh. Physical examination revealed a relatively well-circumscribed nodule, measuring 25×20 mm in diameter, with uneven pigmentation in the patients thigh. Systemic surveillance failed to identify additional tumorous lesions other than the tumor in the left thigh. Total resection of the nodule was performed and subsequently, dissection of the sentinel lymph node. Written informed consent was obtained from the patient.

### Materials and methods

The formalin-fixed, paraffin-embedded tissue blocks of the resected skin specimen and lymph nodes were cut into 3-μm-thick sections, deparaffinized and rehydrated. Each section was stained with hematoxylin and eosin and then used for immunostaining. Immunohistochemical analyses were performed using an autostainer (BenchMark XT system; Ventana Medical System, Tucson, AZ, USA) according to the manufacturer’s instructions. The following primary antibodies were used: Mouse monoclonal antibodies against α-internexin (2E3; Lab Vision Corp., Fremont, CA, USA), cytokeratin (AE1/AE3 and CAM5.2; DakoCytomation, Glostrup, Denmark and Becton-Dickinson, Franklin Lakes, NJ, USA, respectively), glial fibrillary acid protein (GFAP; 6F2; DakoCytomation), HMB-45 (Novocastra Laboratories, Ltd., Newcastle upon Tyne, UK), Melan-A (A103; Novocastra Laboratories, Ltd.), nestin (10C2; Santa Cruz Biotechnology Inc., Santa Cruz, CA, USA), peripherin (PJM50) and vimentin (VIM3B4; both Novocastra Laboratories, Ltd.); and rabbit polyclonal antibodies against S-100 protein (Nichirei Biosciences Inc., Tokyo, Japan) and mTOR (7C10), 4E-BP1 (53H11) and phosphorylated 4E-BP1 (p4E-BP1; Thr 37/46; 236B4) (all Cell Signaling Technology Inc., Danvers, MA, USA).

### Results

**Figure 1:**
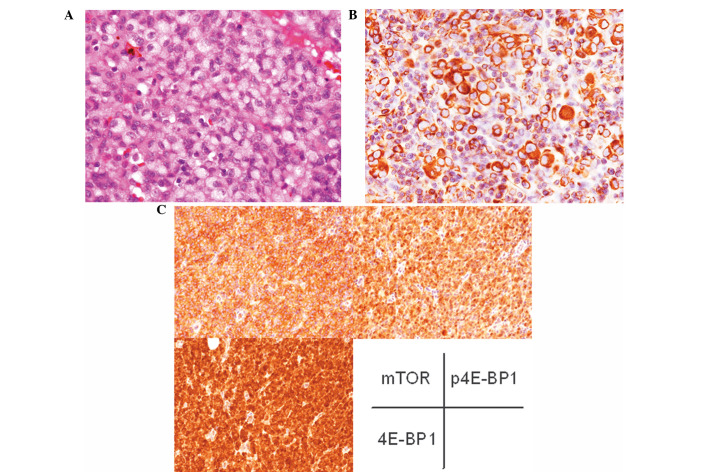
Histopathological and immunohistochemical features of the thigh nodule. (A) Signet-ring cell appearance of the neoplastic cells. The neoplastic cells exhibited eccentrically located enlarged nuclei and abundant pale cytoplasm (hematoxylin and eosin stain; magnification, ×400). (B) Vimentin was expressed in the signet-ring cells (magnification, ×400). (C) mTOR, 4E-BP1 and p4E-BP1 were diffusely expressed (magnification, ×100). mTOR, mammalian target of rapamycin; p4E-BP1, phosphorylated 4E-BP1.

Histopathological examination of the resected thigh nodule revealed proliferation of sheet-like or variable-sized nests composed of medium-sized, round to oval neoplastic cells from the entire dermis to the superficial subcutis. The majority of the neoplastic cells exhibited slightly eosinophilic cytoplasm and centrally located enlarged nuclei with conspicuous nucleoli and specific cells exhibited melanin pigment within the cytoplasm. In the lower section of the lesion, the neoplastic cells showing signet-ring cell appearance were identified (Fig. 1A). These cells exhibited eccentrically located enlarged nuclei with or without conspicuous nucleoli and abundant pale cytoplasm (Fig. 1A). Melanin pigment was not notably observed in the cytoplasm of the signet-ring cells. Mitotic figures were occasionally identified in the entire lesion (6/10 high-power fields). In addition, no atypical melanocytes were observed in the overlying epidermis. Immunohistochemically, the tumor cells, including signet-ring cells, were diffusely positive for S-100 protein, vimentin and Melan-A (Fig. 1B), but negative for HMB-45, cytokeratin (AE1/AE3 and CAM5.2), nestin, peripherin, α-internexin and GFAP. Moreover, mTOR, 4E-BP1 and p4E-BP1 were diffusely expressed in the tumor cells (Fig. 1C).

**Figure 2:**
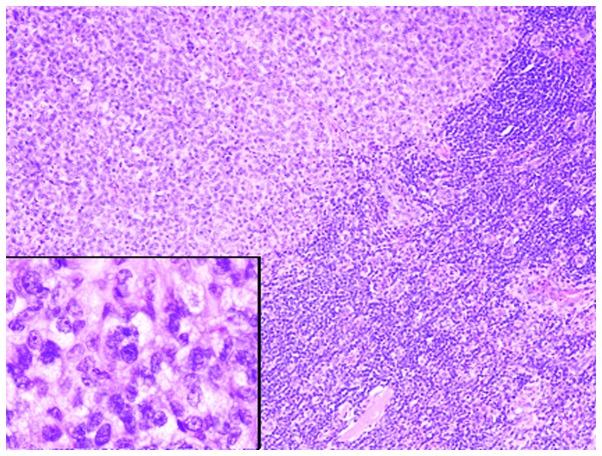
Histopathological features of the sentinel lymph node. Metastatic malignant melanoma was identified. Specific tumor cells showed signet-ring cell appearance as observed in the inset (magnification, ×400) (hematoxylin and eosin stain; magnification, ×100).

The sentinel lymph node exhibited a metastatic malignant melanoma with signet-ring cell component (Fig. 2). Immunohistochemical features of the metastatic melanoma were identical to those of the skin.

According to these histopathological and immunohistochemical features, an ultimate diagnosis of signet-ring cell melanoma with sentinel lymph node metastasis was made.

## Discussion

In cutaneous neoplasms, the presence of signet-ring cells have been reported in a variety of neoplasms, including melanocytic nevi, malignant melanoma, squamous and basal cell carcinoma, hidradenoma and malignant lymphoma (5). Ultrastructural examination revealed that this characteristic morphology in signet-ring cell melanoma is often imparted by the intracytoplasmic accumulation of intermediate filaments, particularly vimentin (2). This observation corroborates the immunohistochemical results of the present case report since vimentin was found to be expressed in the cytoplasm of the signet-ring cells, although, ultrastructural examination was not performed. In addition, analyses of the expression profiles of other intermediate filaments in the signet-ring cell melanoma were not performed. The present case report clearly demonstrated that intermediate filaments, including cytokeratin, peripherin, α-internexin, GFAP and nestin, were not expressed in the signet-ring cell melanoma, although, peripherin and nestin are commonly expressed in malignant melanoma (16). These results indicate that intracytoplasmic accumulation of vimentin, but not other types of intermediate filaments, contributes to the development of the characteristic morphology of signet-ring cell melanoma.

Table I summarizes the clinicopathological features of the 20 previously reported cases of signet-ring cell melanoma as well as the present case. This disease commonly affects middle-aged males (average age of 57.8-years and male/female ratio of 14:7), however, young individuals may also be affected (range, 18–85-years-old). Metastatic signet-ring cell melanoma in a patient with an unknown primary tumor has been documented (2) and six cases, including the present case, exhibited no primary sites (Table I). The present case exhibited no *in situ* component in the overlying epidermis and the patient had no past history of malignant melanoma and tumorous lesions with the exception of the tumor in the thigh, therefore, the cutaneous lesion may not be confirmed as the primary lesion.

Metastatic signet-ring cell melanoma may be a diagnostic issue (4) as it has been reported that signet-ring cells are occasionally present only in metastatic sites and the signet-ring cell melanoma component occasionally lacks melanin pigments in the cytoplasm. Immunohistochemical analyses are useful for generating a correct diagnosis. This type of tumor usually shows positive immunoreactivity for melanocytic markers, including S-100 protein, Melan-A and HMB-45. However, it is important to recognize that exceptions to this phenotype exist as S-100 protein-negative (1/20 cases) or HMB-45-negative cases (4/21 cases) have been documented (Table I) (2,4). Therefore, a combination of these markers is useful for establishing a diagnosis.

In addition, the current case report is the first to analyze the expression profiles of mTOR pathway proteins in signet-ring cell melanoma. mTOR is a central protein involved in carcinogenesis, since it phosphorylates 4E-BP1 which leads to cell proliferation, cell cycle progression and angiogenesis. It has been previously reported that the mTOR pathway is activated in malignant melanomas in contrast to benign melanocytic nevi. Therefore, the mTOR inhibitor is hypothesized to represent a promising therapeutic agent for various types of carcinomas. Specific clinical studies with regard to the mTOR inhibitor have been performed in malignant melanoma (17). The present case study demonstrates that mTOR pathway proteins are activated in signet-ring cell melanoma. Therefore, the mTOR inhibitor may be a potential candidate for the treatment of this type of tumor.

## Figures and Tables

**Table I:** Clinicopathological features of signet-ring cell melanoma.

Case no.	Age, years	Gender	Site	Primary	S-100protein	HMB-45	Vimentin	Author
1	35	Male	Right axillary lymph node	Unknown	+	+	+	Sheibani and Battifora
2	63	Female	Inguinal lymph node	Skin	+	+	+	Sheibani and Battifora
3	57	Female	Lung	NA	−	+	+	Bonetti *et al*
4	55	Male	Arm (recurrence)	Arm	+	+	+	Nakhleh *et al*
5	40	Male	Thigh (recurrence)	Thigh	+	+	+	Nakhleh *et al*
6	80	Male	Left leg	NA	+	+	+	Al-Talib *et al*
7	27	Male	Multiple metastases	Unknown	+	+	+	Eckert *et al*
8	84	Male	Right forearm	Unknown	+	+	NA	LiVolsi *et al*
9	33	Female	Axillary lymph node	Arm	+	−	NA	LiVolsi *et al*
10	85	Female	Skin and inguinal lymph node	Left foot	+	+	NA	LiVolsi *et al*
11	56	Male	Left ear	NA	+	+	NA	LiVolsi *et al*
12	84	Male	Inguinal lymph node	Righ foot	+	+	+	Tsang *et al*
13	55	Male	Abdomen	Unknown	+	+	+	Won *et al*
14	55	Male	Peritoneal effusion	Abdomen	+	+	+	Niemann *et al*
15	72	Female	Left arm	NA	+	+	+	Breier *et al*
16	18	Female	Inguinal lymph node	NA	NA	+	+	Bastian *et al*
17	61	Female	Right shoulder	Right shoulder	+	−	+	Rutten *et al*
18	76	Male	Anterior chest	Anterior chest	+	+	+	Rutten *et al*
19	69	Male	Left shoulder	Left shoulder	+	−	+	Kacerovska *et al*
20	41	Male	Supraclavicular	Unknown	+	+	NA	Russo *et al*
Present	68	Male	Left thigh	Unknown	+	−	+	Ishida *et al*

NA, not available.
